# A multi-mics exploration of programmed cell death in non-obstructive azoospermia: identifying TLR4 as a central regulator and therapeutic target

**DOI:** 10.3389/fcell.2026.1742608

**Published:** 2026-02-06

**Authors:** Qi Yu, Qingtao Yang, Shiwang Yuan, Yili Zhao, Wei Li, Jun Qiao, Fa Sun, Tao Li

**Affiliations:** 1 Department of Urology, The Affiliated Hospital of Guizhou Medical University, Guiyang, China; 2 Department of Reproductive Center, Affiliated Hospital of Guizhou Medical University, Guiyang, China

**Keywords:** male infertility, non-obstructive azoospermia, programmed cell death, spermatogenic dysfunction, TLR4

## Abstract

**Background:**

Male infertility (MI) is a globally recognized public health challenge, affecting approximately 18% of men of reproductive age worldwide. Non-obstructive azoospermia (NOA) is a major cause of NOA and is associated with dysregulated programmed cell death (PCD). However, the precise role and mechanisms of PCD in the pathogenesis of NOA remain poorly understood.

**Methods:**

In this study, target genes associated with both PCD and NOA were retrieved from multiple public databases. Gene Ontology (GO) and Kyoto Encyclopedia of Genes and Genomes (KEGG) enrichment analyses were then performed to explore underlying mechanisms. Subsequently, protein-protein interaction (PPI) network analysis identified hub genes within the network. Mendelian randomization (MR) analysis was further conducted to establish a causal relationship between key genes and NOA susceptibility. Thereafter, *in vitro* cell and molecular biology experiments validated the impact of the pathogenic gene on LPS-induced GC-1 spg (ts) cell injury. Finally, we queried the Comparative Toxicogenomics Database (CTD) to identify environmental exposures and natural bioactive products targeting the pathogenic gene, followed by molecular docking analysis to confirm their interactions.

**Results:**

Our analysis identified 150 PCD-related genes that were dysregulated in NOA. GO and KEGG enrichment analyses indicated that these targets primarily regulate cell death, senescence, inflammation, oxidative stress, and various biosynthetic processes. PPI analysis identified 10 hub genes: HIF1A, TLR4, MDM2, GPX4, SNCA, MTOR, CSNK2A2, ATG5, CTSS, and PIK3CA. Subsequent MR analysis established TLR4 as being causally associated with an increased risk of NOA. *In vitro* experiments confirmed the involvement of TLR4 in LPS-induced damage to GC-1 spg (ts) cells. Finally, CTD database screening and molecular docking analyses identified 8 common environmental pollutants and 9 natural active products that potentially target TLR4, thereby influencing the initiation and progression of NOA.

**Conclusion:**

This study advances the understanding of PCD in the pathogenesis of NOA. It identifies and underscores the critical role of the core PCD-related gene TLR4 in NOA development, highlighting the necessity for strategies aimed at mitigating its negative impact on fertility.

## Introduction

Male infertility (MI) is a globally recognized public health challenge, affecting approximately 18% of men of reproductive age worldwide. Comprehensive analyses indicate that over the past 50 years, the average sperm concentration in Western populations has declined by 52.4%, reflecting a significant global downward trend (Eisenberg et al., 2023; Brannigan et al., 2024; Levine et al., 2023). Beyond its reproductive implications, severe MI is associated with an increased risk of chronic comorbidities, including cardiovascular diseases, autoimmune disorders, and malignancies such as testicular and prostate cancers (Choy and Eisenberg, 2018; Murshidi et al., 2020). Therefore, continued research into the risk factors and pathogenic mechanisms of NOA remains crucial.

Azoospermia, the complete absence of sperm in the ejaculate, is a key etiology of NOA and can be classified as obstructive (OA) or non-obstructive (NOA). NOA accounts for approximately 10% of infertile men, with a prevalence of about 1% in the general male population, making it the most common clinical form of azoospermia (Tang et al., 2023; Takeshima et al., 2024). Its pathogenesis is complex, involving genetic, developmental, endocrine, environmental, and other multifactorial contributors, posing significant challenges for clinical management (Chiba et al., 2016). With the advancement of testicular sperm extraction (TESE) techniques, TESE combined with intracytoplasmic sperm injection (ICSI) has become the primary strategy for NOA patients to achieve biological parenthood (Tang et al., 2023; Corona et al., 2019). However, due to the high heterogeneity of NOA, the success rate of sperm retrieval via TESE is only about 50%. Consequently, in-depth exploration of the pathogenesis of NOA and the identification of relevant molecular markers are of great importance for improving clinical diagnosis and treatment.

Programmed cell death (PCD) is a genetically regulated process essential for normal development and tissue homeostasis (Newton et al., 2024). At least 18 distinct modes of PCD have been identified, including apoptosis, necroptosis, pyroptosis, ferroptosis, cuproptosis, and other regulated cell death pathways (Newton et al., 2024; Liu et al., 2025). Emerging evidence links dysregulated PCD to the pathogenesis of NOA (Piechka et al., 2024; Liu et al., 2023; Dong et al., 2023; Jaiswal et al., 2015). When disrupted by pathological conditions or environmental exposures, these molecular mechanisms can lead to spermatogenic failure, resulting in dysregulation of the testicular microenvironment and subsequent NOA (Piechka et al., 2024; Liu et al., 2023; Dong et al., 2023; Jaiswal et al., 2015). Restoring balanced PCD represents a promising therapeutic strategy for NOA (Piechka et al., 2024). However, a current limitation in the field is the narrow focus on individual PCD pathways, which may overlook complex regulatory networks and subtle yet critical molecular targets.

This study employed testicular tissue microarrays and comprehensive bioinformatics approaches to identify PCD alterations in NOA. Protein-protein interaction analysis was used to identify hub genes, followed by Mendelian randomization (MR) analysis to establish causal relationships between these genes and the risk of developing NOA. Subsequently, the Human Protein Atlas (HPA) database was utilized to determine the expression localization of the causal gene. Furthermore, we conducted *in vitro* functional assays to evaluate the impact of the causal gene on spermatogonia and Sertoli cells. Finally, molecular docking simulations were performed to verify the effects of common environmental exposures on the causal gene and to screen for potential therapeutic natural compounds. Collectively, this work elucidates pathogenic mechanisms and identifies promising therapeutic targets for the management of NOA.

## Methods

### Data sources

Gene expression profile datasets related to NOA were obtained from the Gene Expression Omnibus (GEO) database (http://www.ncbi.nlm.nih.gov/geo/), specifically accessions GSE4797, GSE45885, GSE45887, and GSE145467. A combined dataset (GSE_combined) was created by merging GSE45885 and GSE45887 using the “sva” R package function to correct for batch effects. This combined dataset was used for differential expression analysis to identify disease-related targets. GSE4797 and GSE145467 were used as independent cohorts to validate the expression of key genes.

### Acquisition of programmed cell death-related genes associated with non-obstructive azoospermia

Genes associated with 18 PCD subtypes were identified through a comprehensive search of the MSigDB, GeneCards, and KEGG databases, along with a review of relevant literature. The PCD subtypes included: apoptosis, pyroptosis, ferroptosis, autophagy, necroptosis, cuproptosis, parthanatos, endogenous cell death, eosinophil cell death, lysosome-dependent cell death, alkaliptosis, oxytosis, neutrophil extracellular trap-associated cell death, immunogenic cell death, anoikis, paraptosis, large-scale apoptotic cell death, and intracellular engulfment-related cell death. Differential expression analysis was performed on the integrated GSE_combined dataset using the “Limma” R package with thresholds of |log2FC| > 0.65 and an adjusted p-value <0.05 to identify NOA-related targets. Venn analysis was subsequently used to reveal dysregulated PCD-related genes in NOA patients, representing the imbalanced PCD pathways involved in NOA pathogenesis.

### Functional enrichment analysis

Gene Ontology (GO) categorizes gene function into Cellular Component (CC), Molecular Function (MF), and Biological Process (BP) domains. The Kyoto Encyclopedia of Genes and Genomes (KEGG) systematically link genomic information with higher-level functional pathways. GO and KEGG enrichment analyses were performed using the R package clusterProfiler.

### Construction of protein-protein interaction (PPI) network

The STRING database (confidence score threshold ≥0.4), which integrates known and predicted protein associations including physical interactions and functional links, was used to analyze interactions among the overlapping targets. After retrieving PPI data, non-essential targets were filtered out to construct a target-protein interaction network. This interaction network (in TSV format) was imported into Cytoscape 3.9.0 for visualization. Centiscape2.2, a plugin within Cytoscape, was used to calculate topological properties (Degree, Closeness, Betweenness) to assess node importance. Topological analysis using “CentiScape 2.2”identified hub targets based on three centrality measures: Closeness Centrality ≥0.00281, Betweenness Centrality ≥240.538, and Degree Centrality ≥7.415.

### Mendelian randomization analysis

This study employed a two-sample Mendelian randomization (MR) analysis to infer a potential causal relationship between genetically predicted gene expression and the risk of male infertility (MI). This methodological approach relies on three core instrumental variable (IV) assumptions: (1) Relevance–the genetic IVs must be robustly associated with the exposure (gene expression level); (2) Independence–the IVs must not be associated with any known or unknown confounders of the exposure-outcome relationship; and (3) Exclusion Restriction–the IVs must influence the outcome solely through the exposure, with no alternative biological pathways. To obtain valid IVs adhering to these assumptions, stringent criteria were applied. Genetic instruments for gene expression (exposure) were selected as cis-expression quantitative trait loci (cis-eQTLs) from the eQTLGen Consortium, a resource integrating data from 31,684 individuals predominantly of European ancestry (Kurki et al., 2023). Single-nucleotide polymorphisms (SNPs) were retained at a genome-wide significance threshold (p < 5 × 10^−8^) and clumped (*r* ^2^ < 0.001, window size = 10,000 kb) to ensure independence, with palindromic SNPs removed. The strength of each IV was quantified using the F-statistic (F > 10 indicated sufficient strength to minimize weak instrument bias). Summary statistics for the outcome (MI) were sourced from the FinnGen Release 12 genome-wide association study, comprising 1,852 cases and 153,573 controls, all of European ancestry (Võsa et al., 2021). The primary causal estimate was derived using the Inverse-Variance Weighted (IVW) method.

### Sensitivity analysis of mendelian randomization analysis

To critically evaluate the validity of the MR assumptions and the robustness of findings, a suite of sensitivity analyses was performed: (1) Cochran’s Q test assessed heterogeneity across IVs (with a random-effects IVW model applied if P < 0.05); (2) the MR-Egger intercept test detected directional horizontal pleiotropy, a violation of the independence and exclusion restriction assumptions; and (3) leave-one-out analysis verified that the causal estimate was not disproportionately driven by any single influential SNP. A significant causal association was concluded if the IVW P-value was <0.05, supported by consistent results from sensitivity analyses indicating no substantial pleiotropy or heterogeneity.

## Validation and localization of key genes

To clarify the cellular localization and function of key genes, immunohistochemistry data and single-cell RNA sequencing data were retrieved from the Human Protein Atlas (HPA) database to determine the localization patterns of key genes within human testicular tissue.

### Cell culture and treatment

The GC-1 spg (ts) mouse spermatogonial cell line was obtained from the Cell Bank of the Chinese Academy of Sciences. Cells were cultured according to the manufacturer’s protocol at 37 °C in a humidified atmosphere containing 5% CO_2_. The culture medium was high-glucose Dulbecco’s Modified Eagle Medium (DMEM), and cells were seeded at a density of 1 × 10^5^ cells. The complete growth medium was supplemented with 10% fetal bovine serum (FBS). To investigate the role of TLR4 in non-obstructive azoospermia (NOA), an inflammatory model was established based on previous literature. Cells were treated with 1 μg/mL lipopolysaccharide (LPS; Sigma, L2880) for 12 h to mimic testicular inflammation associated with human infertility. Subsequently, to verify the specific role of TLR4, this inflammatory model was treated with 10 μM TAK-242, a specific TLR4 inhibitor, as reported in prior studies. Similarly, the intervention doses for the selected compounds, perfluorooctanoic acid (PFOA) and baicalein, were derived from established literature. The final concentrations used were 500 μM for PFOA and 20 μM for baicalein (Biswas and Yenugu, 2013; Ye et al., 2019; Luo et al., 2025; Wan et al., 2018; Chen et al., 2021).

### Western blot analysis

Total protein was extracted using RIPA lysis buffer (Solarbio, China) supplemented with a protease inhibitor cocktail (Yeasen, China). Protein concentration was quantified using the Pierce™ BCA Protein Assay Kit (Thermo Fisher Scientific). Amounts of protein (4–6 µg per lane) were separated on 8%–12% gradient SDS-PAGE gels and transferred to PVDF membranes using a semi-dry transfer system (Bio-Rad). Membranes were blocked with 5% skim milk in TBST for 1 h at room temperature, then incubated overnight at 4 °C with primary antibodies. The primary antibodies used were anti-TLR4 (1:2,000, # 19811-1-AP, Proteintech), anti-NF-κB p65 (1:500, # 10745-1-AP, Proteintech) and anti-GAPDH (1:50,000, # 60004-1-Ig, Proteintech). After washing, incubate the membrane with HRP conjugated anti rabbit IgG secondary antibody at room temperature. Finally, visualize the protein bands and use Image Lab ™ Quantitative analysis was performed using Bio Rad software.

#### EdU proliferation assay

The Cell-Light™ EdU Apollo® 488 *In Vitro* Kit (Beyotime Biotechnology, Shanghai) was used for proliferation analysis. GC-1 spg (ts) cells were seeded in 24-well plates (3 × 10^4^ cells/well) and cultured until 70%–80% confluent. Cells were pulse-labeled with 20 μM EdU working solution (Servicebio, China) for 2 h in a 37 °C, 5% CO_2_ incubator. After fixation with 4% paraformaldehyde (PFA) for 15 min, cells were permeabilized with 0.5% Triton X-100 for 20 min. Proliferating cells were stained using the Click-iT™ EdU Alexa Fluor™ 594 Imaging Kit (Thermo Fisher Scientific) according to the manufacturer’s protocol. Fluorescence images were captured using an inverted fluorescence microscope (Nikon Eclipse Ts2R FL) at ×20 magnification.

### Identification of potential environmental exposures and natural active products

The Comparative Toxicogenomics Database (CTD) was utilized to identify small molecule compounds targeting the key gene, with a focus on common environmental exposures and natural bioactive products. Following initial screening, molecular docking simulations were performed to validate compound-target interactions. The molecular structures of environmental exposures and natural products were retrieved from PubChem. The three-dimensional structure of the key target protein was obtained from the AlphaFold Protein Structure Database. AutoDockTools 1.5.7 was used for structure preparation and docking simulations to predict binding modes, binding affinity (ΔG), and functional implications. A binding energy <0 kcal/mol indicates spontaneous binding, while an energy <−5.0 kcal/mol suggests a stable interaction.

## Result

### Identification of programmed cell death related genes associated with non-obstructive azoospermia (NOA-PCD)

**FIGURE 1:**
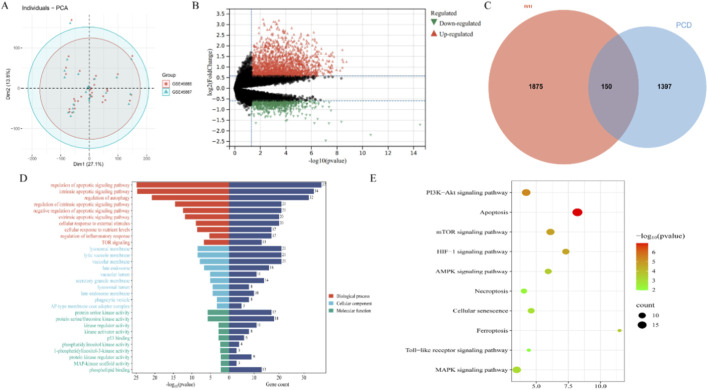
Acquisition and enrichment analysis of PCD-NOA overlapping genes. **(A)** PCA Plot After Batch Effect Removal; **(B)** Volcano Plot of Differentially Expressed Genes in NOA Patients; **(B)** PCD-NOA genes; **(D)** GO enrichment analysis results; **(E)** KEGG enrichment analysis results.

Database integration identified 1,964 PCD-related genes across 18 subtypes: apoptosis (580), pyroptosis (52), ferroptosis (88), autophagy (367), necroptosis (101), cuproptosis (19), parthanatos (9), netosis (15), lysosome-dependent death (220), alkaliptosis (7), oxytosis (5), NETosis (24), immunogenic cell death (34), anoikis (338), paraptosis (66), mitotic catastrophe (8), and entosis (23). Complete details are provided in Supplementary Table S1. Batch effect correction was applied to the merged dataset (Figure 1A), yielding an integrated training set comprising 8 control and 43 NOA samples. Differential expression analysis identified 2,025 significant DEGs (1,484 downregulated and 541 upregulated in NOA versus controls) (Figure 1B). Venn analysis subsequently identified 150 dysregulated PCD-related genes, representing pathogenic PCD regulators in NOA (Figure 1C).

### Enrichment analysis

GO enrichment analysis of the 150 NOA-PCD genes revealed their significant involvement in crucial biological processes, including positive and negative regulation of apoptotic signaling pathway, regulation of autophagy, cellular response to nutrient levels, cellular response to extracellular stimuli, and regulation of inflammatory response (Figure 1D). KEGG pathway analysis further identified pathways closely associated with cell proliferation and programmed cell death, such as the PI3K-Akt signaling pathway, mTOR signaling pathway, HIF-1 signaling pathway, ferroptosis, cellular senescence, necroptosis, apoptosis, Toll-like receptor signaling pathway, and MAPK signaling pathway (Figure 1E). These findings collectively suggest that dysregulated PCD may contribute to NOA pathogenesis through multiple distinct pathways.

### Protein-protein interaction network of non-obstructive azoospermia-programmed cell death targets

**FIGURE 2:**
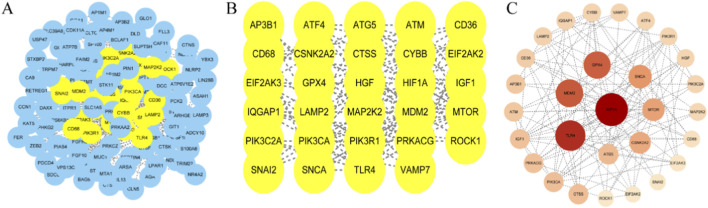
PPI interaction network of PCD-NOA gene and screening process of core genes. **(A)** PPI interaction network of PCD-NOA gene; **(B)** PCD-NOA core genes; **(C)** PCD-NOA core genes sorted by degree.

The 150 overlapping NOA-PCD target genes were imported into the STRING database for PPI analysis, applying a confidence threshold of ≥0.4. After filtering disconnected nodes, 143 NOA-PCD target proteins remained in the final network. The PPI network was visualized using Cytoscape. Nodes were arranged using a degree-based layout, where darker colors and larger diameters indicate stronger protein interactions (Figure 2A). Topological analysis identified 10 hub genes within the NOA-PCD interaction network: HIF1A, TLR4, MDM2, GPX4, SNCA, MTOR, CSNK2A2, ATG5, CTSS, and PIK3CA (Figures 2B,C). This visualization provides a clear overview of the interactions between key targets and offers valuable insights for further investigation into the molecular mechanisms linking PCD and NOA.

### Association between hub genes and male infertility risk

**FIGURE 3:**
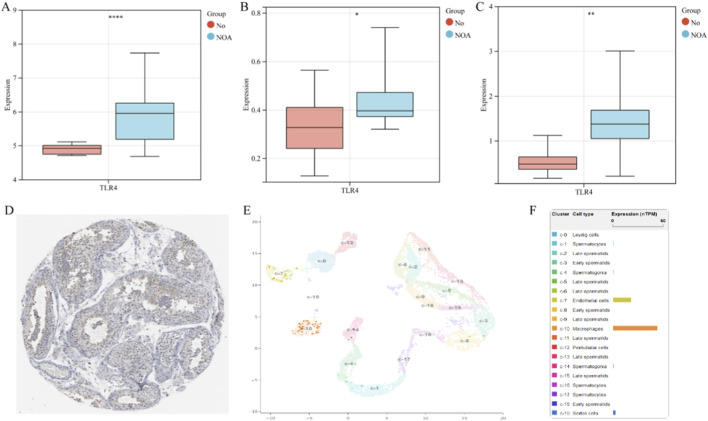
Expression of TLR4 **(A–C)**: The expression of TLR4 in three NOA cohorts; **(D)**: The expression of TLR4 in testicular tissue displayed by HPA; **(E)**: The expression of TLR4 in single-cell subpopulations of testes.

After excluding the core gene ATG5 due to a lack of valid instrumental variables, a two-sample MR analysis was performed on the remaining nine candidate genes. The results (Table 1) demonstrated a significant causal relationship between genetically proxied TLR4 expression and an increased risk of MI (Odds Ratio [OR]: 1.16; 95% Confidence Interval [CI]: 1.01–1.33; *p* = 0.032). In contrast, the other eight candidate genes showed no significant association with MI susceptibility (all *p* > 0.05). Sensitivity analyses confirmed the robustness of the primary finding for TLR4 (Table 1). Specifically, the MR-Egger intercept test indicated no evidence of directional pleiotropy (*p* = 0.66), Cochran’s Q test showed no significant heterogeneity (*p* = 0.99), and leave-one-out analysis demonstrated that the causal estimate was consistent and not driven by any single influential genetic variant. Consistent with the MR results, TLR4 expression was found to be significantly upregulated in NOA samples across discovery queue (GSE) (Figure 3A) and independent validation cohorts (GSE4797 and GSE145467) (Figures 3B,C). Based on this converging evidence, TLR4 was selected as the primary focus for subsequent analyses.

**TABLE 1:** Causal relationship between serum levels of core genes and increased risk of male infertility.

Gene	Method	NSNP	OR	LCI95	UCI95	P_MR	P_ple	P_het
MTOR	WR	1.00	0.82	0.66	1.01	0.06	NA	NA
CTSS	IVW	8.00	1.02	0.86	1.21	0.80	0.79	0.05
SNCA	IVW	9.00	1.04	0.87	1.24	0.65	0.22	0.67
TLR4	IVW	6.00	1.16	1.01	1.33	0.04	0.66	0.99
MDM2	IVW	5.00	0.97	0.85	1.10	0.61	0.36	0.41
GPX4	IVW	4.00	0.99	0.89	1.11	0.92	0.51	0.55
CSNK2A2	WR	1.00	0.87	0.35	2.15	0.76	NA	NA
HIF1A	IVW	4.00	1.18	0.90	1.54	0.23	0.46	0.29
PIK3CA	IVW	2.00	0.74	0.46	1.18	0.20	NA	0.26

WR, wald ratio; IWV, inverse variance weighted; OR, odds ratio; P_MR, *P*-value of Mendelian randomization analysis; P_ple, *P*-value of horizontal pleiotropy suggestion; P_het, *P*-value of heterogeneity test.

### Validation and localization of TLR4 expression

TLR4 expression was assessed using integrated datasets, immunohistochemistry data, and single-cell RNA sequencing data from the Human Protein Atlas (HPA) database. Our analysis revealed that TLR4 is primarily expressed within the seminiferous tubules, with lower expression levels detected in other testicular cellular compartments (Figure 3D). Consistent with this spatial localization, single-cell transcriptomic data demonstrated that in healthy testicular tissue, TLR4 expression is predominantly enriched in macrophages and endothelial cells. Moderate expression was observed in Sertoli cells, while low-level expression was detected in spermatocytes and spermatogonia. TLR4 expression was scarcely detectable in Leydig cells, peritubular cells, as well as in early and late spermatids (Figures 3E,F).

### Impact of TLR4 on the GC-1 spg (ts) inflammatory injury cell model

**FIGURE 4:**
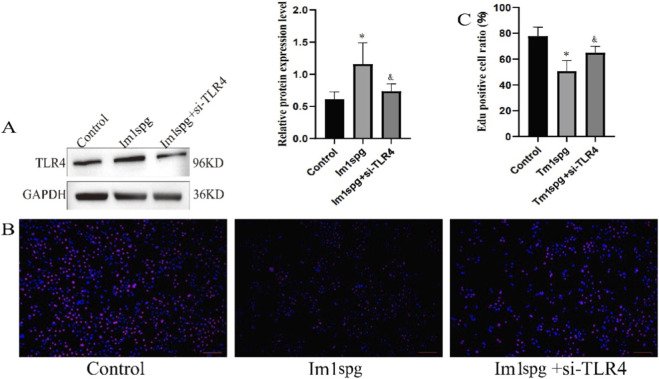
The effect of TLR4 on GC-1 spg (ts) cells **(A)** TLR4 expression status; **(B)** Fluorescence image of Edu experiment; **(C)** Edu positive cell ratio, Scale bar: 100 µm. Note: */& p < 0.05 Vs. Im1spg.

To further evaluate the role of TLR4 in the context of NOA, we established an LPS-induced inflammatory injury model using GC-1 spg (ts) spermatogonial cells (designated as the Im1spd model) to simulate the inflammatory microenvironment of NOA, following established methodology. Western blot analysis confirmed significantly increased TLR4 expression in the Im1spd model compared to control cells (Figure 4A). Subsequent functional assays demonstrated that TLR4 inhibit effectively attenuated the inflammatory damage in GC-1 spg (ts) cells: EdU incorporation assays revealed significantly protected proliferative capacity against inflammation-induced injury (Figures 4B,C). Collectively, these data highlight TLR4 as a key mediator in the pathogenesis of NOA.

#### Effects of common environmental pollutants on TLR4

**FIGURE 5:**
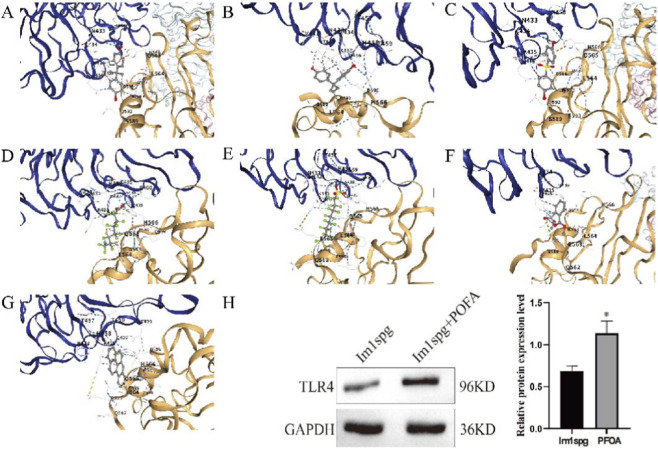
The impact of seven common environmental pollutants on TLR4. **(A)** BPA; **(B)** BPB; **(C)** BPS; **(D)** PFOA; **(E)** PFOS; **(F)** DMP; **(G)** BaP; **(H)** The effect of PFOA on TLR4 expression in Im1spg model. Note: *p < 0.05 Vs. Im1spg.

To identify common environmental pollutants targeting TLR4, we queried the CTD. This search identified eight such pollutants associated with TLR4: bisphenol A (BPA), bisphenol B (BPB), bisphenol S (BPS), perfluorooctanoic acid (PFOA), perfluorooctanesulfonic acid (PFOS), dimethyl phthalate (DMP), and benzo[a]pyrene (BaP). Subsequent molecular docking simulations confirmed that all eight compounds could bind stably to TLR4, with binding energies (ΔG) below −5 kcal/mol. The calculated binding energies were as follows: BPA (−7.3 kcal/mol; Figure 5A), BPB (−6.9 kcal/mol; Figure 5B), BPS (−7.4 kcal/mol; Figure 5C), PFOA (−8.4 kcal/mol; Figure 5D), PFOS (−5.3 kcal/mol; Figure 5E), DMP (−8.2 kcal/mol; Figure 5F), and BaP (−8.2 kcal/mol; Figure 5E). To validate the molecular docking predictions, Western blot analysis revealed that treatment with PFOA, the compound with the highest predicted binding affinity, significantly upregulated the LPS-induced expression of both TLR4 and its downstream effector NF-κB p65 in GC-1 spg (ts) cells (Supplementary Figure S1A), and exacerbated LPS-induced proliferation arrest (Supplementary Figures S1B,C). In summary, these findings suggest that TLR4 is a key molecular target through which environmental pollutants may act to participate in the pathogenesis of NOA.

### Potential bioactive therapeutic agents

**FIGURE 6:**
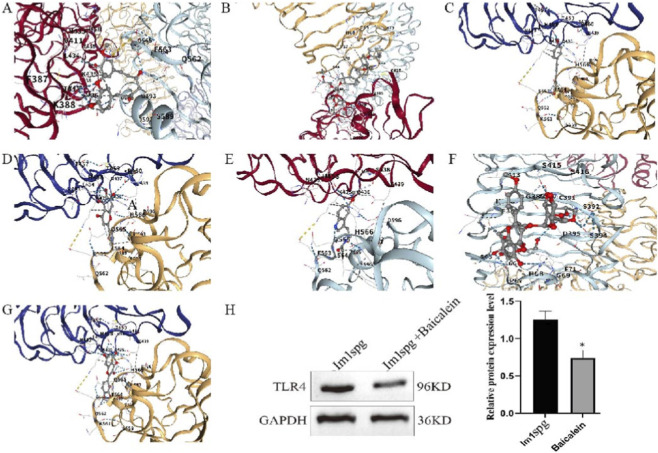
The effects of seven natural active products on TLR4. **(A)** curcumin; **(B)** ginsenoside Rg3; **(C)** resveratrol **(D)** baicalein; **(E)** melatonin; **(F)** icariin B; **(G)** quercetin; **(H)** The effect of baicalein on TLR4 expression in Im1spg model. Note: *p < 0.05 Vs. Im1spg.

To identify potential natural bioactive compounds for the treatment of NOA, we focused on TLR4-associated natural products compiled in the CTD. Our screening identified seven common natural active compounds related to TLR4: curcuNOAn, ginsenoside Rg3, resveratrol, melatonin, baicalein, icariin B, and quercetin. These compounds are well-known for their notable pharmacological properties, including anti-inflammatory, antioxidant, and anti-apoptotic activities. Subsequent molecular docking analysis confirmed that all seven natural products could bind stably to TLR4, with binding energies (ΔG) below −5 kcal/mol. The specific binding energies were as follows: curcumin (−8.4 kcal/mol; Figure 6A), ginsenoside Rg3 (−8.1 kcal/mol; Figure 6B), resveratrol (−7.8 kcal/mol; Figure 6C), baicalein (−9.2 kcal/mol; Figure 6D), melatonin (−7.1 kcal/mol; Figure 6E), icariin B (−8.1 kcal/mol; Figure 6F), and quercetin (−9.0 kcal/mol; Figure 6G). Consistent with our computational predictions, Western blot analysis confirmed that baicalein treatment significantly reduced the LPS-induced expression of both TLR4 and NF-κB p65 (Figure 6H; Supplementary Figure S1C) and alleviated LPS-induced proliferation arrest (Supplementary Figures S1B,C). Together, these findings highlight the potential of natural bioactive compounds such as baicalein in developing novel therapeutic strategies for NOA.

## Discussion

Over the past 4 decades, the sustained decline in male fertility has become a major global concern. Beyond contributing to reduced population growth, this trend profoundly impacts patients’ psychological wellbeing, quality of life, and family relationships, while imposing substantial economic and emotional burdens (Eisenberg et al., 2023; Brannigan et al., 2024; Levine et al., 2023; Choy and Eisenberg, 2018; Murshidi et al., 2020). The pathogenesis of MI is complex, with NOA being a primary factor. Unlike obstructive azoospermia patients with normal spermatogenesis, NOA patients frequently exhibit varying types and severities of spermatogenic failure. In addition to genetic predisposition and aging, environmental and lifestyle factors are recognized as key determinants of risk (Tang et al., 2023; Takeshima et al., 2024; Chiba et al., 2016). Recent research has increasingly focused on the role of PCD in NOA, suggesting that multiple PCD mechanisms may critically contribute to defects in spermatogenesis (Newton et al., 2024; Liu et al., 2025; Piechka et al., 2024; Liu et al., 2023; Dong et al., 2023; Jaiswal et al., 2015); however, the precise relationship between PCD dysregulation and NOA pathogenesis remains unclear. Continued investigation into PCD dysregulation in NOA may help elucidate disease mechanisms and identify potential therapeutic targets.

In this study, we employed a multidisciplinary approach, integrating bioinformatics, MR, molecular docking, and *in vitro* cellular assays to investigate potential PCD-NOA links, based on transcriptomic data from NOA patients and gene sets associated with 18 PCD subtypes. Our analysis identified 150 hub genes linking PCD and NOA pathways. We further characterized key genes and their interaction networks, providing novel insights into the role of PCD in NOA pathogenesis. KEGG and GO enrichment analyses suggested that PCD dysregulation in NOA patients might contribute to disease progression by modulating inflammatory responses, oxidative stress, cell death pathways, cellular senescence, DNA damage, and biosynthetic processes. PPI network analysis identified ten core regulatory genes: HIF1A, TLR4, MDM2, GPX4, SNCA, MTOR, CSNK2A2, ATG5, CTSS, and PIK3CA. Finally, MR analysis established a causal relationship between TLR4 and genetic susceptibility to NOA.

TLR4 is a transmembrane receptor that plays a central role in innate immune responses. Upon activation by exogenous stimuli or tissue damage, it initiates defense mechanisms (Kim et al., 2023; Oda et al., 2024). However, excessive TLR4 activation disrupts immune homeostasis by sustaining the production of pro-inflammatory cytokines and chemokines, thereby contributing to the development of various diseases, including Alzheimer’s disease, cancer, and osteoarthritis (Heidari et al., 2022; Zhang et al., 2013; Shetab Boushehri and Lamprecht, 2018; Oo, 2024). Previous studies have confirmed the critical role of dysregulated immune homeostasis in NOA pathogenesis (Chen et al., 2016; Zhao et al., 2014). In the testes, this dysregulation can be triggered by conditions such as varicocele, obesity, genital infections, leukocytospermia, physical obstruction or trauma, and exposure to toxic substances (Li et al., 2024). Due to prolonged or untreated inflammation, testicular resident cells responsible for maintaining spermatogenesis suffer DNA damage, lipid and protein oxidation, and mitochondrial dysfunction. This leads to functional loss in affected Sertoli and Leydig cells and the formation of morphologically abnormal, dysfunctional sperm cells (Chen et al., 2016; Zhao et al., 2014; Li et al., 2024; Kang et al., 2021; Ye et al., 2021), forming the basis of NOA. Consequently, maintaining immune homeostasis within the testes of NOA patients is crucial for its prevention and treatment.

Accumulating evidence underscores the role of TLR4 in the male reproductive system (Mansouri et al., 2025; Chen et al., 2024). Studies have confirmed its expression in the mammalian male reproductive tract, including the testes, epididymis, vas deferens, and accessory glands (Fujita et al., 2011). This suggests that under pathological conditions, excessive activation of TLR4 could disrupt immune homeostasis in the testicular microenvironment, leading to NOA. For instance, in diabetic patients, hyperglycemia impairs testicular microcirculation and hemodynamics, inducing oxidative stress. Excess reactive oxygen species (ROS) further activate TLR4, amplifying inflammation and oxidative stress (Karpova et al., 2020; Naas et al., 2019). Similarly, studies have identified TLR4 as a downstream target of environmental pollutants (e.g., microplastics, copper, cadmium, furans) and reproductive toxicants (e.g., cisplatin, gentamicin) (Akhigbe et al., 2024; Hagan et al., 2015; Xia et al., 2024). Exposure to these substances upregulates TLR4 activity and expression, disrupting testicular immune homeostasis, which impairs the function of Sertoli and Leydig cells, promotes the formation of morphologically abnormal sperm, and ultimately leads to NOA (Akhigbe et al., 2024; Hagan et al., 2015; Xia et al., 2024). Conversely, certain beneficial agents ameliorate external factor-induced testicular injury by targeting TLR4 (Mao et al., 2021; Hu et al., 2024). Collectively, this experimental evidence highlights the pivotal role of TLR4 in NOA pathogenesis and its potential as a therapeutic target.

Finally, to advance prevention and treatment strategies for NOA, our study identified two key pathways mediated by TLR4: Pathogenic Pathway:7 common environmental pollutants, BPA, BPB, BPS, PFOA, PFOS, DMP, and BaP, may contribute to NOA pathogenesis by targeting TLR4. Therapeutic Pathway: 7 natural bioactive compounds, curcumin, ginsenoside Rg3, resveratrol, baicalein, melatonin, icariin B, and quercetin. These findings significantly expand our understanding of environmental risk factors for NOA and highlight promising candidates for therapeutic development targeting the TLR4 pathway.

Compared to existing studies, this work provides a more systematic examination of the role of PCD in NOA by incorporating a broader range of PCD subtypes and integrating multi-omics data with diverse bioinformatics approaches. However, we acknowledge several limitations in the current study. First, heterogeneity in the genetic data, such as differences in sequencing platforms and the racial backgrounds of the populations, may have influenced the analytical outcomes. Second, while the importance of TLR4 was validated through multi-omics strategies and external data, the limited sample size may still introduce bias, necessitating further confirmation in larger-scale cohort studies. Furthermore, although we employed a well-established LPS-induced *in vitro* inflammatory model, the field of NOA currently lacks a universally accepted cellular disease model.

Additionally, while we preliminarily validated TLR4 as a common target for environmental pollutants and natural products, only a single pollutant and a single natural compound were investigated. Their direct regulatory mechanisms remain unclear and warrant further validation through future studies employing approaches such as domain-specific mutagenesis, reporter gene assays, and *in vivo*/*in vitro* models. Furthermore, the drug concentrations used in this study were based on doses reported in previous literature, and detailed dose-response curves were not established. While the selected doses allowed us to achieve the primary objectives of this mechanistic study, comprehensive dose-response analyses are essential for defining therapeutic windows and pharmacological efficacy. Future studies should therefore include systematic dose-response profiling. Finally, this work was conducted primarily in the GC-1 spg (ts) cell line, which represents only one specific stage of spermatogenesis. The stage-specific regulatory roles of TLR4 across different germ cell types remain to be elucidated. Subsequent research using primary germ cells or spermatogenic co-culture systems will be crucial for systematically revealing the dynamic role of TLR4 in the pathogenesis of NOA.

## Conclusion

This study employed an integrated approach combining bioinformatics, molecular docking, and *in vitro* cellular assays to comprehensively investigate dysregulated PCD in the testicular tissue of NOA patients. Our findings indicate that dysregulated PCD influences the initiation and progression of NOA through multiple molecular targets and signaling pathways. We provide the first demonstration of an association between TLR4 expression and susceptibility to NOA, further corroborated by *in vitro* experiments confirming TLR4’s involvement in LPS-induced damage to testicular Sertoli cells. Furthermore, we identified novel environmental pollutants that may exacerbate NOA and natural bioactive compounds with therapeutic potential for NOA. Overall, this work provides new mechanistic insights into PCD dysregulation in NOA pathogenesis. Future studies should employ comprehensive *in vitro* and *in vivo* models to further validate the pathophysiological impact of PCD dysregulation on NOA.

## Data Availability

The original contributions presented in the study are included in the article/Supplementary Material, further inquiries can be directed to the corresponding authors.

## Supplementary material

SUPPLEMENTARY FIGURE S1 The impact of PFOA and baicalein on the Im1spg model A: The impact of PFOA and baicalein on NF-κB68 expression; B-C: The effects of PFOA and baicalein on the proliferation of Im1spg model, Scale bar: 100 µm. Note: */& *p* < 0.05 Vs. Im1spg.
