# Supplementary material for: A multi-mics exploration of programmed cell death in non-obstructive azoospermia: identifying TLR4 as a central regulator and therapeutic target
Source: Front Cell Dev Biol. 2026 Feb 6;14:1742608. doi: 10.3389/fcell.2026.1742608 (PMC12920511; doi:10.3389/fcell.2026.1742608)
Supplement: Supplementary file 1 [file DataSheet1.docx]

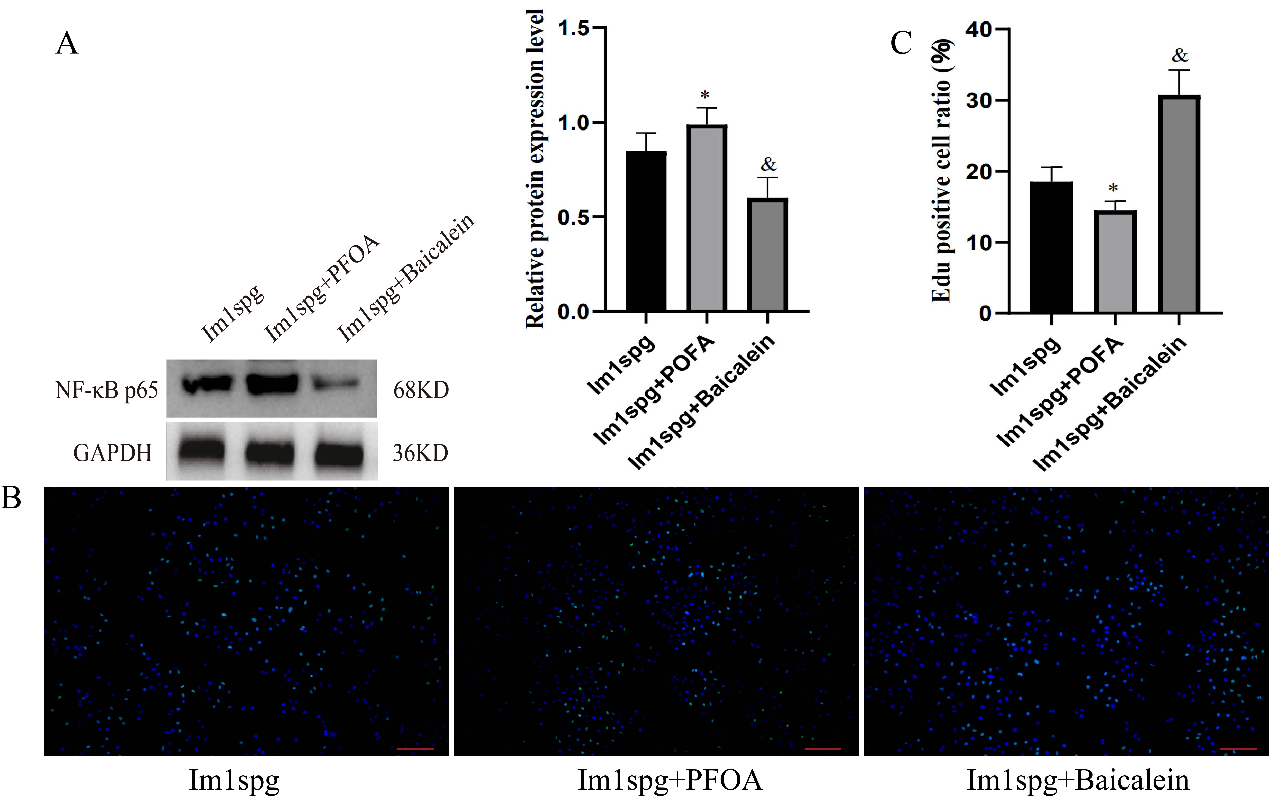


Fig S1: The impact of PFOA and baicalein on the Im1spg model

A: The impact of PFOA and baicalein on NF-κB68 expression; B-C: The effects of PFOA and baicalein on the proliferation of Im1spg model, Scale bar: 100 µm.
